# Influence of antibiotics on bacterial load and sperm parameters during short-term preservation of collared peccary semen

**DOI:** 10.1590/1984-3143-AR2021-0021

**Published:** 2021-09-13

**Authors:** Caio Sérgio Santos, Lívia Batista Campos, Érica Camila Gurgel Praxedes, Samara Sandy Jerônimo Moreira, João Batista Freire Souza-Júnior, Pierre Comizzoli, Francisco Marlon Carneiro Feijó, Alexandre Rodrigues Silva

**Affiliations:** 1 Laboratório de Conservação de Germoplasma Animal, Universidade Federal Rural do Semi-Árido – UFERSA, Mossoró, RN, Brasil; 2 Smithsonian Conservation Biology Institute, National Zoological Park, Washington D.C., United States of America; 3 Laboratório de Microbiologia Veterinária, Universidade Federal Rural do Semi-Árido – UFERSA, Mossoró-RN, Brasil

**Keywords:** antibiotics, extenders, semen, peccary, short-term storage

## Abstract

Studies on semen and sperm cells are critical to develop assisted reproductive technologies for the conservation of the collared peccary. The objective of the study was to compare the effect of different antibiotics on the bacterial load and sperm quality during short-term storage of peccary semen. Fresh semen samples from 10 males were extended in Tris-egg yolk or Tris-*Aloe vera* supplemented with streptomycin-penicillin (SP; 1 mg/mL - 1000 IU/mL or 2 mg/mL - 2000 IU/mL) or gentamicin (30 µg/mL or 70 µg/mL) before storage at 5°C. Bacterial load and sperm motility, membrane integrity and function, mitochondrial activity, and morphology, were evaluated at different time points for 36 h. The SP and gentamicin treatments concentration inhibited (*p* < 0.05) bacterial growth for 36 h regardless of the extender. Compared to the other treatments, Tris-egg yolk plus 70 µg/mL gentamicin maintained the sperm parameters for longer, including total motility (41.9 ± 6.1%) at 24 h, and membrane integrity (58.3 ± 2.1%) at 36 h. In contrast, the highest SP concentration in both extenders impaired sperm membrane integrity at 36 h (*p* < 0.05). For the liquid storage of collared peccary semen, it therefore is recommended to use Tris extender supplemented with egg yolk and gentamicin (70 µg/mL).

## Introduction

The collared peccary (*Pecari tajacu*) is a wild ungulate distributed across the American continents, having important ecological roles as seed dispersers, and serving as prey for large carnivores (Desbiez et al., 2012). Although their population has been relatively stable worldwide (Gongora et al., 2011), it has been declining in some biomes in the recent years as a consequence of anthropic actions that destroy the habitats and fragment the areas occupied by these animals (Desbiez et al., 2012). As a consequence, several studies in assisted reproductive techniques have been conducted, especially regarding short-term preservation protocols of semen (Garcia et al., 2012; Souza et al., 2016). This is an important research area because optimal short-term preservation allows the exchange of genetic material between distant regions for conservation or commercial breeding peccaries (Garcia et al., 2015).

However, risks of pathogen and disease transmissions are associated with semen exchange between facilities for artificial insemination, (Eaglesome and Garcia, 1997). As part these efforts, our team recently described the semen microbiome in peccaries, including the presence of *Staphylococcus sp.* and, specially, *Corynebacterium sp*., whose high amounts have been shown to impair some peccary sperm parameters as membrane integrity and curvilinear velocity (Santos et al., 2020). Similarly, certain types of bacteria can directly impair sperm parameters and thus interfere with fertility in swine (Maroto-Martín et al., 2010), the domestic species most closely related to the peccaries (Bosma et al., 2004). Current literature reports that boar semen could be contaminated with more than 3.5 × 10^3^ CFU/ml *Escherichia coli* (alone or associated to other Gram negative bacteria), which can cause sperm agglutination and reduce litter size in inseminated sows (Maroto-Martín et al., 2010). The effects on sperm cells such as agglutination, decreased motility, damage to the acrosome and membrane integrity, which also interfere with sperm storage longevity, depend on the types and load of bacterial isolates (Kuster and Althouse, 2016). Another interesting point is that in domestic boars, Gram negative bacteria are mainly described, such as *Stenotrophomonas maltophilia*, *Alcaligenes xylosoxidans*, *Serratia marcescens*, *Escherichia coli*, *Pseudomonas* spp., *Enterobacter cloacae* and others (Althouse et al., 2000; Althouse and Lu, 2005). On the other hand, we identified various Gram positive bacteria in peccary semen, as *Staphylococcus* sp., *Arcanobacterium* sp., *Bacillus* sp. and *Dermabacter* sp. (Santos et al., 2020), which highlight some differences in the composition of reproductive microbiome among different species.

Antibiotics are usually added to semen extenders to prevent bacterial multiplication or kill bacteria (Morrell and Wallgren, 2011). This is an important step to prevent the spread of microorganisms through the use of semen samples in assisted reproductive technologies. (Santos and Silva, 2020). However, some antibiotics have been reported to negatively affect sperm quality in different species in a dose dependent manner (Santos and Silva, 2020). Overall, antibiotics that are most used in the composition of extenders for semen preservation in cattle (Almquist, 1951) and horses (Dean et al., 2012) include a combination of streptomycin and penicillin. In swine, however, gentamicin has been established as the main antimicrobial in semen extenders (Schulze et al., 2017). For wild animals, studies describing the use of antimicrobial drugs in the preservation of semen are scarce (Johnston et al., 1998).

For peccaries, short-term preservation of semen was achieved at the use of a Tris-based extenders, constituted by different external cryoprotectants as the egg yolk or the Aloe vera gel (Souza et al., 2016). Therefore, the objective of the study was to compare the effect of different antibiotics on the bacterial load and sperm quality during short-term storage of peccary semen using different extenders.

## Material and methods

### Ethical considerations

All experimental procedures were approved by the Animal Use Ethics Committee of the Federal Rural University of Semi-arid – UFERSA (No. 23091.009851/2018-96), and by the Chico Mendes Institute for Biodiversity Conservation (No. 37329/3).

### Animals

The study was conducted with animals from the Center for Wild Animals Multiplication (CEMAS) of UFERSA, which is located in Mossoró, a Brazilian semi-arid region (5º10´S-37º10´W; average temperature range, 27 - 29ºC) and registered as a scientific breeding center (IBAMA No. 1478912). Ten sexually mature males (mean age 40 months) were used for the study. The animals were exposed to natural outdoor photo period (~12 h) and segregated into groups of five in paddocks (20 m × 3 m) with covered area of 6 m^2^. Animals were fed an isocaloric (3 300 kcal/kg) and isoproteic (14% protein) diet consisting of corn (79.8%), soybean meal (15.4%), wheat bran (1.45%), calcium (2.6%), and a vitamin (0.2%) and mineral premix (0.05%), supplemented with tropical fruits, such as melon. Water was provided *ad libitum*.

### Experimental design

The samples were obtained from 10 individuals. After obtaining the ejaculates, one aliquot of fresh semen was immediately evaluated, and other 10 aliquots were used for short-term preservation. Two of these aliquots were diluted in Tris-based extenders containing egg yolk or *Aloe vera* gel, as the control groups without antibiotics. The other eight aliquots were diluted with the same Tris-based extenders containing the streptomycin-penicillin combination (Sigma, Sigma-Aldrich, São Paulo, Brazil) at a concentration of 2 mg/mL-2000 IU/mL or 1 mg/mL-1000 IU/mL, or containing the gentamicin (Gentatec®, Chemitec®, São Paulo, Brazil) at 70 or 30 µg/mL. These concentrations were chosen based on in vitro sensitivity tests that were previously performed against the main isolates in fresh semen from collared peccaries (Santos et al., 2020).

### Semen collection

The animals were fasted for 12 h prior to the semen collection procedure. They were restrained with a hand net and anesthetized through intravenous administration of propofol (Propovan^®^, Cristália, Fortaleza, Brazil) in bolus (5 mg/kg) (Souza et al., 2009). During the procedure, a venous catheter was introduced into the cephalic vein for fluid therapy with 0.9% physiological saline solution and the vital signs were monitored. Before semen collection, genitalia of the animals were washed with physiologic saline solution.

Semen was collected using an electroejaculator (Autojac®, Neovet, Campinas, São Paulo, Brazil), connected to a 12 V source. The electroejaculator probe measured 15 cm (length) and 1.3 cm (diameter); and 12 cm were inserted into the rectum of the male. The stimulation cycle consisted of ten stimuli at each voltage, starting at 5 V, followed by a 1 V increase to 12 V. Each electrical stimulus lasted 3 seconds, with intermittent intervals of 2 seconds. The stimulation cycle lasted 10 minutes (Alves et al., 2013). Semen samples were collected in sterile plastic tubes and divided into two aliquots. One of the aliquots was immediately evaluated for bacterial load, and the other was subjected to sperm analysis.

### Bacterial quantification

Semen processing began with the inoculation of 100 µl aliquots of each sample into 900 µl of 0.85% sterile saline (dilution 10^-1^) followed by serial dilution to 10^-5^. Then, 100 µl aliquots of each dilution were plated with a Drigalski handle on the surface of Petri dishes containing Plate Count Agar (Hi Media, Mumbai, India), which is indicated for enumeration of mesophilic aerobic bacteria that encompass several genera isolated in biological and environmental samples. All samples were tested in triplicates and plates were incubated in a bacteriological incubator (Fanem LTDA, São Paulo, Brazil) at 37º C for 24 - 48 h. Following incubation, the colonies were counted, and the average number of bacteria was expressed as CFU per milliliter multiplied by the inverse of each dilution (Tortora et al., 2017).

### Semen analysis

The semen was evaluated for volume, appearance, color, and pH immediately after collection. The volume was measured using automatic micropipettes; the appearance and color were subjectively observed; and the pH was measured using pH strips evaluated according to the color scale. To determine the sperm concentration, a 10 µl aliquot of the semen was diluted in 2 ml of buffered formaldehyde solution (10%) and analyzed using a Neubauer counting chamber (Souza et al., 2016).

Sperm kinetic parameters were evaluated using an automated IVOS 7.4G system (Hamilton-Research^TM^ Thorne, Beverly, MA, USA) using the settings previously established for the species (Souza et al., 2016). The following parameters were evaluated: total motility (%), velocity average pathway (VAP, µm/s), velocity straight line (VSL, µm/s), velocity curvilinear (VCL, µm/s), amplitude lateral head (ALH, µm), beat cross frequency (BCF, Hz), straightness (STR, %), and linearity (LIN, %) as well as the sperm subpopulations: rapid, medium, slow, and static.

The hypo-osmotic test was performed to assess the functional integrity of the sperm cell membrane using distilled water (0 mOsm/L) as a hypo-osmotic solution. For morphological analysis of the sperm, semen smears were stained with Bengal Rose (Sigma-Aldrich, St. Louis, USA) and observed under a light microscope (×1000; 200 cells/slide) (Souza et al., 2016).

For membrane integrity and mitochondrial potential evaluation, a semen aliquot (10 μL) was incubated at 37 °C for 10 min in a solution composed of the following combination of fluorescent probes: 2 μL Propidium Iodide (PI, Sigma-Aldrich, St. Louis, USA), 5 μL CMXRos (Mito Tracker® Red, Invitrogen®, Oregon, USA), and 3 μL Hoechst 33342 (Sigma-Aldrich, St. Louis, USA). Next, the samples were evaluated with an epifluorescence microscope (Episcopic Fluorescent Attachment “EFA” Halogen Lamp Set, Leica, Kista, Sweden). A total of 200 spermatozoa (per sample) were evaluated for the plasma membrane integrity using PI/H342 association and for mitochondrial membrane potential through CMXRos fluorescence. The sperm heads marked in blue were considered to possess intact membranes and those totally or partially marked in red were considered to be not intact; sperms with regions of the midpiece marked in red were considered as presenting mitochondrial activity (Souza et al., 2016).

### Semen storage

Immediately after collection, semen aliquots were diluted according to the experimental design. The streptomycin-penicillin combination and the gentamicin, at different concentrations were added to the Tris-citrate-fructose extender supplemented with either of two external cryoprotectants, egg yolk (Alves et al., 2013) and *Aloe vera* gel (Souza et al., 2016) at a 20% concentration. All groups were adjusted to the same sperm concentration (100 × 10^6^ sperm/mL).

After dilution, samples were stored in the water jacket at 27 ºC and equilibrated for 40 minutes to reach 15 ºC in a biological incubator (Quimis, Diadema, SP, Brazil). Furthermore, the incubator was adjusted to establish a temperature at 5 ºC for 30 minutes. Every 12 hours, semen aliquots were rewarmed at 37 ºC and reevaluated for bacterial load and sperm parameters, as previously described, till 36 hours.

### Statistical analysis

Sperm characteristics and bacterial concentration are expressed as mean ± Standard Error (SE). The data were first examined for normality using the Shapiro - Wilk test and for homoscedasticity using Levene’s test. Data were transformed by log (x + 1) or arc-sine (√ (x/100)), when necessary, to meet the assumptions (or assumptions) of the parametric analysis. A two-way ANOVA using a general linear model using the PROC GLM procedure of the Statistical Analysis System (SAS Institute Inc.) was performed to evaluate the effects of the treatment, incubation time (0, 12, 24, and 36 h) and its interaction on the studied parameters. Tukey post-hoc test was used to verify the potential differences between the means. Statistical significance was set at *P* < 0.05.

## Results

### Fresh semen evaluation

Samples had a whitish color and aqueous appearance, with a pH of 7.4 ± 0.2. The average volume was 1.9 ± 0.4 ml with sperm concentration of 379 ± 40.7 × 10^6^ sperm/ml. The average value for total motile sperm was 79.4 ± 3.0%, with 83.1 ± 1.6% sperm with intact membranes and 72.2 ± 3.4% with osmotic response. In addition, an average of 77.7 ± 2.8% morphologically normal sperm and 82.8 ± 1.3% mitochondrial activity were found. A bacterial load of 2.3 ± 0.9 × 10^6^ CFU/mL was found in fresh semen samples.

### Impact of semen extenders and antibiotics on bacterial load

**Figure 1:**
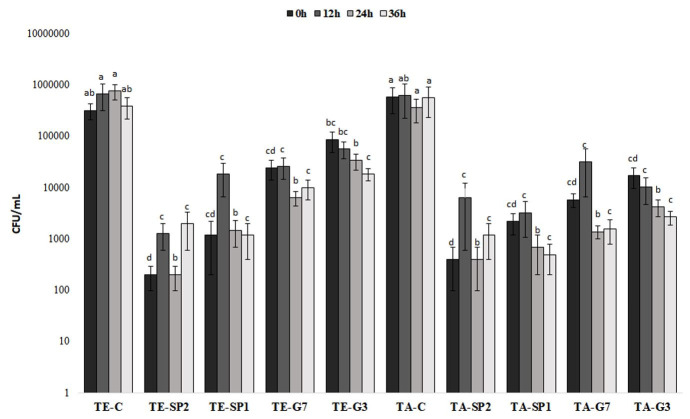
Values (mean ± SEM) for bacterial load (CFU/mL) in collared peccaries (*Pecari tajacu –* n = 10) semen following short-term preservation for 36 h and dilution in Tris-egg yolk (TE) and Tris-*Aloe vera* (TA) with and without different antibiotics concentrations (control – C, 2 mg/mL-2000 IU/mL of streptomycin-penicillin – SP2, 1 mg/mL-1000 IU/mL of streptomycin-penicillin – SP1, 70 µg/mL of gentamicin – G7 and 30 µg/mL of gentamicin – G3) during chilling for 36h. ^a-d^ Lowercase letters indicate significant differences for treatments at the same time (*p* < 0.05). No differences were found between storage times.

The bacterial loads observed during semen storage are shown in Figure 1. The results demonstrated that both streptomycin-penicillin and gentamicin at any concentration, controlled the bacterial load during the entire storage period of 36 h. On the other hand, samples diluted in Tris supplemented with only egg yolk or *Aloe vera*, without antibiotics, failed to control bacterial growth during semen storage.

Regardless of the extenders used, only samples containing streptomycin-penicillin combination at either of the two concentrations eliminated 100% of the bacterial load for 36 h in the samples of four (Tris plus egg yolk) and three (Tris plus *Aloe vera*) individuals. For the other individuals, antibiotics were not able to fully eliminate the microorganisms.

### Impact of semen extenders and antibiotics on sperm motion

From a biological standpoint, evaluation of the sperm motility and kinetic parameters (Table 1) revealed that samples diluted in Tris-egg yolk supplemented of streptomycin-penicillin at 1 mg/mL-1000 IU/mL or gentamicin at 70 µg/mL maintained (*p* < 0.05) progressive motility for long periods (only declining at 36 h). At general, treatments diluted in Tris egg-yolk, regardless the use of antibiotics, provided more efficient (*p* < 0.05) preservation of total and progressive sperm motility compared to the treatments diluted in *Tris-Aloe* vera with or without antibiotics during storage for 36 h.

**Table 1:** Values (mean ± SEM) for sperm motility kinetic parameters in semen samples collared peccaries (*Pecari tajacu* – n = 10) exposed to different treatments. Semen was chilled at 5 °C with Tris-egg yolk and Tris-*Aloe vera* supplemented and without antibiotics and assessed at different time points up to 36h. Antibiotics concentrations were streptomycin-penicillin at 2000 IU/mL (SP2) or 1 mg/mL-1000 IU/mL (SP1), or the gentamicin at 70 (G7) or 30 (G3) µg/mL.

**Parameters**	**Time**	**Groups**									
		**Tris-egg yolk**					**Tris-Aloe vera**				
		**Control**	**SP2**	**SP1**	**G7**	**G3**	**Control**	**SP2**	**SP1**	**G7**	**G3**
Total motility (%)	0h	66.1 ± 4.4 ^A^	50.3 ± 5.4 ^A^	61.8 ± 4.3 ^A^	63.5 ± 5.5 ^A^	63.7 ± 5 ^A^	50.2 ± 9.1 ^A^	61.8 ± 4.3 ^A^	66.9 ± 6.6 ^A^	58.7 ± 7.1 ^A^	58.2 ± 7.9 ^A^
	12h	49.9 ± 6.9^aAB^	28 ± 6.4^abAB^	42.8 ± 6.2^aAB^	47.2 ± 5.7^aAB^	35.7 ± 5.7^abAB^	9.6 ± 4.2^cB^	13.3 ± 4^bcB^	12.4 ± 4.9^bcB^	11.7 ± 3.3^bcB^	12.6 ± 2.7^bcB^
	24h	42.8 ± 7.3^aAB^	15.3 ± 4.4^acB^	34.7 ± 6.2^aAB^	41.9 ± 6.1^aAB^	34.1 ± 8.2^abAB^	8.7 ± 2.9^bcB^	5.4 ± 1.2^cB^	7.7 ± 2.3^bcB^	6.8 ± 1.5^bcB^	4.9 ± 1.4^cB^
	36h	30.7 ± 6.2^aB^	9.1 ± 2.4^abB^	25.6 ± 5.9^abB^	20.3 ±5.5^abB^	26.3 ± 4.8^abB^	7.8 ± 3^bB^	6 ± 2.7^bB^	4.4 ± 0.8^bB^	6.8 ± 3.7^bB^	16.9 ± 4.3^bB^
Progressive motility (%)	0h	36.8 ± 4.9 ^A^	23.1 ± 4.7 ^A^	32.7 ± 6.7 ^A^	35.9 ± 5.8 ^A^	37.4 ± 5.9 ^A^	17 ± 4.7 ^A^	23.9 ± 6.7 ^A^	25.2 ± 7.8 ^A^	24.4 ± 5.4 ^A^	22.8 ± 6.9 ^A^
	12h	15.6 ± 1.9^aAB^	6.5 ± 1.6^abAB^	14.2 ± 2.2^aAB^	18.1 ± 2.6^aAB^	9.6 ± 1.3^abB^	1 ± 0.4^bB^	2.5 ± 1^bB^	2.3 ± 1.2^bB^	2.1 ± 0.9^bB^	2.3 ± 0.4^bB^
	24h	13.5 ± 2.5^aB^	3.1 ± 1^abB^	10.7 ± 2.1^abB^	15 ± 2.6^aAB^	10.6 ± 3.1^abB^	1.7 ± 0.7^bB^	1 ± 0.3^bB^	1.1 ± 0.5^bB^	1.5 ± 0.4^bB^	1.2 ± 0.4^bB^
	36h	11.8 ± 2.9^aB^	2.2 ± 0.9^abB^	6.4 ± 2.2^abB^	7.2 ± 2.1^abB^	9.2 ± 1.7^abB^	1 ± 0.4^bB^	1.1 ± 0.4^abB^	1 ± 0.4^abB^	1.7 ± 1^abB^	0.5 ± 0.2^bB^
Average path velocity (µm/s)	0h	51.4 ± 3.4	38.5 ± 0.9	45.6 ± 2	46.2 ± 1.8	50.3 ± 2.5	38 ± 3.5	38.9 ± 1.9	40.4 ± 2	42.7 ± 2.5^A^	39.3 ± 2.2^A^
	12h	44.4 ± 1.7^ab^	36 ± 3.3^ac^	44.3 ± 2.8^ab^	48 ± 3.6^a^	44.8 ± 2.3^ab^	28.5 ± 2.4^c^	31.2 ±3.4b^c^	28.4 ± 1.7^c^	31 ± 1.5^bcAB^	32.6 ± 1.8^bcAB^
	24h	45.7 ± 2.7^ab^	32.2 ± 2.1^bcde^	40.3 ± 2.2^abcd^	47.8 ± 1.9^a^	41.4 ± 3.8^abc^	31.9 ± 1.5^bcde^	30.5 ± 1.7^cde^	26.2 ± 3.5^de^	30.3 ± 2.5^cdeAB^	24.1 ± 4.3^eB^
	36h	39.8 ± 2.7^ac^	28.9 ± 4.1^ac^	33.8 ± 1.5^ac^	40 ± 3.4^ab^	41.5 ± 2.1^a^	25.4 ±1.8^c^	28.8 ± 2.7^ac^	27.2 ± 3.7^ac^	25.8 ± 1.6^bcB^	25.9 ± 1.7^bcB^
Straight line velocity (µm/s)	0h	30.6 ± 1.5	23.7 ± 0.9	29.5 ± 2^A^	28 ± 1.7	30.1 ± 1.3	21.1 ± 1.4	21.8 ± 1.4	22.4 ± 1.8	22.9 ± 1.2	21.4 ± 1.8
	12h	23.6 ± 1.4^ab^	19.9 ± 2.2^ab^	24 ± 1.9^abAB^	27.3 ± 2.6^a^	23.3 ± 1.7^ab^	14.2 ± 1.4^b^	14.8 ± 1.4^b^	16.6 ± 1.1^b^	17.2 ± 1.3^ab^	17.6 ± 1^ab^
	24h	25 ± 1.6^ab^	17.4 ± 1.6^ac^	21.8 ± 1.6^acAB^	26.6 ± 1.3^a^	21.8 ± 2.6^ac^	18.1 ± 1.4^ac^	19 ± 2.6^ac^	16.1 ± 2.3^bc^	17.3 ± 2.6^ac^	13.5 ± 3.2^c^
	36h	22.7 ± 2.1^a^	16.8 ± 2.6^ab^	17.8 ± 1.5^abB^	22.9 ± 2.2^a^	23.3 ± 1.4^a^	12.3 ± 1.1^b^	19.6 ± 2.5^ab^	14.7 ± 1.3^ab^	15 ± 0.9^ab^	15.9 ± 2.1^ab^
Curvilinear velocity (µm/s)	0h	114.2 ± 5.9^aA^	82.9 ± 1.5^c^	104 ± 6.8^acA^	104.1 ± 3.9^ac^	112.7 ± 4.6^abA^	82.6 ± 6.9^cA^	85.2 ± 4.3^bcA^	86.1 ± 6.1^acA^	93.3 ± 4^acA^	86.2 ± 4.5^acA^
	12h	89.1 ± 3.5^abAB^	66.7 ± 6^ac^	84.6 ± 6.2^acAB^	93.8 ± 6^a^	88.9 ± 6.1^abAB^	56.5 ± 3.5^cAB^	62.2 ± 6.1^bcAB^	56.1 ± 4.5^cB^	57.7 ± 3.6^cB^	64.1 ± 4.4^bcAB^
	24h	89.4 ± 5.3^abAB^	61.2 ± 3.7^bc^	76.1 ± 4.3^acB^	93.4 ± 4^a^	79.2 ± 8.2^abB^	65.6 ± 3.5^acAB^	55.5 ± 2.3^bcB^	49.4 ± 6.5^cB^	57.8 ± 4.4^bcB^	49.7 ± 8.7^cB^
	36h	78.9 ± 4.7^abB^	61.7 ± 4.9^ac^	66 ± 3.6^acB^	76.7 ± 5.8^ab^	80.6 ± 3.8^aB^	51 ± 3.7^bcB^	54.4 ± 4.6^acB^	53.1 ± 7.2^acB^	53.1 ± 5.8^acB^	47.6 ±2.2^cB^
Rapid motility (%)	0h	52.8 ± 4.7 ^A^	33.4 ± 5 ^A^	47.7 ± 5.9 ^A^	50.1 ± 6.3 ^A^	52.7 ± 6.6 ^A^	31.5 ± 6.3 ^A^	37.3 ± 7.6 ^A^	40.5 ± 9.8 ^A^	41.3 ± 6.5 ^A^	35.3 ± 8.2 ^A^
	12h	33.1 ± 4.2^aAB^	14.3 ± 3.7^acAB^	29.4 ± 4.9^abAB^	34.8 ± 4.6^aAB^	23.9 ± 4.1^acB^	2.3 ± 0.8^cB^	6.3 ± 2.4^bcB^	5 ± 2.5^bcB^	5.1 ± 1.8^bcB^	5.6 ± 0.9^bcB^
	24h	29.6 ± 5.6^aAB^	7.6 ± 2.9^abB^	22.8 ± 5.1^abB^	30 ± 4.9^aAB^	23.2 ± 6^abB^	3.8 ± 1.2^bB^	2.1 ± 0.6^bB^	2.6 ± 1^bB^	2.7 ± 0.7^bB^	2.3 ± 0.7^bB^
	36h	22.3 ± 4.9^B^	4.7 ± 1.7^B^	14.8 ± 3.8^B^	14.8 ± 4.4^B^	18.6 ± 3.9^B^	2.2 ± 0.8^B^	2.2 ± 0.7^B^	1.7 ± 0.6^B^	3.1 ± 1.8^B^	1.2 ± 0.4^B^
Static (%)	0h	30.1 ± 4.2	44.5 ± 6 ^A^	34.3 ± 4.6	33.4 ± 5.6 ^A^	32.7 ± 4.9 ^A^	45.3 ± 9.3 ^A^	39 ± 7.5 ^A^	39.9 ± 10.3 ^A^	37.4 ± 7.3 ^A^	42.6 ± 9.7 ^A^
	12h	42.6 ± 7.1^b^	65.8 ± 6.7^abAB^	50.1 ± 6.3^b^	45.8 ± 6.2^bAB^	57.8 ± 6.1^abAB^	87 ± 5.7^aB^	83.7 ± 4.3^aB^	85 ± 5.6^aB^	85.3 ± 3.6^aB^	84.3 ± 2.7^aB^
	24h	50.7 ± 7.9^d^	80.4 ± 5.3^adB^	59.1 ± 6.9^cd^	51.2 ± 6.8^dAB^	61.4 ± 8.9^bdAB^	89.2 ± 3^abcB^	92.6 ± 1.3^abB^	90.3 ± 2.6^abB^	91 ± 2^abB^	93.6 ± 1.8^aB^
	36h	62.6 ± 6.8^b^	87.7 ± 3^abB^	68.6 ± 6.6^ab^	75.2 ± 6.6^abB^	68.9 ± 5.4^abB^	89.3 ± 3.9^abB^	91 ± 3.3^abB^	93.9 ± 0.7^aB^	91.2 ± 4.2^aB^	94.9 ± 1.6^aB^

^a-e^ Values with different lowercase letters in rows differ significantly (*p* < 0.05); ^A-B^ Values with different uppercase letters in columns differs significantly.

Among the other sperm kinetic parameters obtained using CASA (Table 1), all the samples diluted in Tris-egg yolk provided higher average values for VAP (*p* < 0.05) than those diluted in Tris-*Aloe vera*, regardless of the antibiotics used. An important finding regarding the progression of velocities over time was that the treatments Tris-egg yolk plus streptomycin- penicillin at 2000 IU/mL and Tris-egg yolk plus gentamicin at 30 or 70 µg/mL maintained (p > 0.05) the VCL values during storage for 36 h. Regarding sperm subpopulations, the use of Tris-egg yolk supplemented of 70 µg/mL gentamicin resulted in maintenance of the number of fast sperm for up to 24 h just like in the control group; however, for other treatments, a decrease on the percentage of rapid sperm was already observed at 12 h.

Further, data obtained using CASA including amplitude of lateral head (ALH), beat cross frequency (BCF), straightness (STR), linearity (LIN), and medium, and slow motility sperm, showed no relevant or no significant differences.

### Impact of semen extenders and antibiotics on sperm membrane integrity, mitochondrial potential, osmotic response, and morphology

During storage for 36 h, the use of streptomycin-penicillin or gentamicin at any concentration added to the Tris-egg yolk extender provided more efficient preservation (*p* < 0.05) of the sperm membrane integrity and mitochondrial activity compared to all the groups diluted in Tris-*Aloe vera* with or without antibiotics (Table 2). Even in the use of Tris-egg yolk extender, supplementation with the highest concentration of streptomycin-penicillin impaired sperm membrane integrity at 36 h, which did not occur at the gentamicin use (*p* < 0.05). Along the storage time, a decrease on sperm mitochondrial potential was observed with the use of both antibiotics (*p* < 0.05); however, all groups diluted in Tris-egg yolk provided values similar to the control group at each evaluation time, while the use of *Aloe vera* negatively affected the mitochondrial activity (Table 2).

**Table 2:** Values (mean ± SEM) for plasma membrane integrity. mitochondrial activity osmotic response and sperm morphology in collared peccaries (*Pecari tajacu* – n = 10) chilling (5 °C) semen (n=10) diluted in Tris-egg yolk and Tris-Aloe vera with and without different antibiotics concentrations as the streptomycin-penicillin at 2000 IU/mL (SP2) or 1 mg/mL-1000 IU/mL (SP1), or the gentamicin at 70 (G7) or 30 (G3) µg/mL, during refrigeration at 5ºC for 36 h.

**Parameters**	**Time**	**Groups**									
		**Tris-egg yolk**					**Tris-Aloe vera**				
		**Control**	**SP2**	**SP1**	**G7**	**G3**	**Control**	**SP2**	**SP1**	**G7**	**G3**
Sperm membrane integrity (%)	0h	71.9 ± 2	79.1 ± 3.4^A^	79.8 ± 2.5	78.1 ± 2	73.8 ± 2	61 ± 3.5^A^	65.3 ± 5.1^A^	66.9 ± 4^A^	62.5 ± 2.7^A^	56.8 ± 3.6^A^
	12h	64.5 ± 5.4^ab^	68.7 ± 4.9^aA^	66.4 ± 3.1^a^	70.1 ± 3.4^a^	66.2 ± 3.5^a^	40.4 ± 4.3^cAB^	34.7 ± 5.9^cB^	41.5 ± 7.1^bcB^	39.5 ± 5.4^cAB^	39.3 ± 8^cAB^
	24h	60.9 ± 3.5^a^	56.4 ± 4.8^aA^	62.2 ± 3.4^a^	67.5 ± 3.3^a^	60.5 ± 3.2^a^	29 ± 6.6^bB^	27.8 ± 5^bB^	30.6 ± 3.5^bB^	22.6 ± 4.8^bB^	28.5 ± 5.6^bB^
	36h	56.6 ± 3.5^a^	51.1 ± 4.2^aB^	59.3 ± 2.9^a^	58.3 ± 2.1^a^	60 ± 2.6^a^	22.4 ± 4^bB^	23.8 ± 4.4^bB^	24.3 ± 4.6^bB^	24.5 ± 3.8^bB^	20.7 ± 5.1^bB^
Mitochondrial activity (%)	0h	71 ± 3.2	75.9 ± 2.6^A^	80.5 ± 2.8^A^	78.5 ± 1.9^A^	72.3 ± 1.7	60.5 ± 4.4^A^	74.1 ± 3.4^A^	63.9 ± 6.6^A^	66.4 ± 4.5^A^	63.7 ± 4.3 ^A^
	12h	63.3 ± 3.1^a^	64.7 ± 4.3^aAB^	63.5 ± 2.7^aAB^	69 ± 3.2^aAB^	60.8 ± 3.9^a^	37.6 ± 3.3^bAB^	31.1 ± 5.4^bB^	34.1 ± 7.7^bB^	33.2 ± 5.8^bB^	32.4 ± 6^bB^
	24h	56.5 ± 3.1^a^	49.1 ± 4.8^aB^	58.7 ± 3^aAB^	67 ± 3.3^aAB^	58.9 ± 3.4^a^	25 ± 6^bB^	23.6 ± 3.8^bB^	21.9 ± 3.4^bB^	23.1 ± 5.6^bB^	20.8 ± 3.1^bB^
	36h	53.1 ± 2.6^a^	49.8 ± 5.3^aB^	57.3 ± 3.5^aB^	53.6 ± 4.2^aB^	58.1 ± 3.5^a^	17.3 ± 3.6^bB^	20.9 ± 4.5^bB^	22.2 ± 5.1^bB^	17.6 ± 3.6^bB^	16.9 ± 4.3^bB^
Osmotic response (%)	0h	55 ± 5.9	64.9 ± 3.3	60.9 ± 3.4	50.3 ± 4.4	50.6 ± 5.3	52.1 ± 4.9	57 ± 3.7	59.6 ± 5	56.1 ± 3.4	57.3 ± 6.1
	12h	51.3 ± 5.4	60 ± 4.5	56.4 ± 4.1	61.7 ± 3.6	54.8 ± 4.7	48.7 ± 5.9	44.8 ± 4.7	38.2 ± 4.8	45 ± 5.7	42.1 ± 6.4
	24h	56.4 ± 6.5	59 ± 4.3	60.4 ± 5.4	57.1 ± 5.9	61.4 ± 5.9	39 ± 7.1	44.6 ± 6.7	37.1 ± 6.7	37.8 ± 5.8	34.3 ± 6.7
	36h	43.8 ± 6.6	50 ± 6.2	47.5 ± 4.4	43.9 ± 4.9	50.7 ± 4.7	31.9 ± 3.1	41.1 ± 8.1	31.9 ± 5.6	37.7 ± 4.9	35.7 ± 5.4
Normal morphology (%)	0h	74.5 ± 3.8	73.6 ± 3	73.8 ± 3.5	75.2 ± 3.6	75.3 ± 3.9	74.8 ± 3.2	71.5 ± 3.2	72.6 ± 3.7	75.5 ± 3.2	73.4 ± 3.3
	12h	71.2 ± 4	71.1 ± 3.6	73.3 ± 3.5	72.5 ± 3.6	72.5 ± 3.4	72.5 ± 3.8	72.7 ± 3.6	71.4 ± 3.4	72.2 ± 3.6	71.1 ± 3.6
	24h	67.8 ± 3.4	71.2 ± 4.5	71.7 ± 3.9	68.8 ± 4	68.8 ± 4.5	71.2 ± 4.5	72.3 ± 3.3	69.8 ± 4.3	70.8 ± 4.2	67.9 ± 4.2
	36h	68.3 ± 4.8	68.7 ± 4.2	66.1 ± 4.1	70.1 ± 4.5	71.4 ± 5	68.8 ± 3.9	70.8 ± 4.2	69.6 ± 5.3	67.5 ± 4.6	67.8 ± 4.8

^a-b^ Values with different lowercase letters in rows differ significantly (*p* < 0.05); ^A-B^ Values with different uppercase letters in columns differs significantly.

Regarding the preservation of osmotic response and sperm morphology (Table 2), there were no significant differences among treatments or storage times during the semen preservation for 36 h.

## Discussion

The efforts related to the implementation of assisted reproductive technologies applied to wildlife conservation must consider species-specific variations regarding semen physiology (Fickel et al., 2007). Especially with regard to peccaries, our previous studies on seminal biochemistry showed that the proportion of inorganic components, such as calcium and magnesium, differs from studies in the domestic swine (Moreira et al., 2019). Furthermore, the investigation of the seminal plasma proteome of peccaries revealed the presence of a large proportion of clusterin compared to swine semen, in which spermadhesins are more abundant (Santos et al., 2014). Based on these considerations, we understand why the protocols for cryopreservation of collared peccary semen, with regard to freezing curves (Silva et al., 2013) and the proportions of low-density lipoprotein added to the diluent (Souza et al., 2015) differ widely from those used for pigs. However, the scarcity of information regarding the storage of semen of collared peccaries under refrigeration prompts us to carry out further investigations such as the present study, with the aim of establishing efficient protocols for this purpose.

The addition of antibiotics was essential for controlling the bacterial load during short-term storage of peccary semen, since samples diluted in Tris-based extenders without antibiotics failed to control the amount of microorganisms. Besides controlling bacterial load, addition of gentamicin provided the most efficient preservation of motility kinetic parameters and membrane integrity in peccary sperm. Similarly, gentamicin provides effective bacterial control during liquid storage of swine semen (Waberski et al., 2019). Like all aminoglycoside antibiotics, gentamicin blocks the production of protein by binding to the 30S ribosome, thus inhibiting messenger RNA in the bacterial cell (Hahn and Sarre, 1969).

An efficient preservation of sperm progressive motility, VAP, fast subpopulation, membrane integrity and mitochondrial potential were obtained when peccary semen was chilled in the extender containing egg yolk, supplemented with gentamicin, especially at the highest concentration (70 µg/mL). In domestic swine, gentamicin did not interfere with sperm motility parameters during long-term semen storage (6 days at 15°C) and when combined with other drugs such as florfenicol and polimixin B, it improved progressive motility and mitochondrial potential (Bryła and Trzcińska, 2015), even in the use of higher doses of gentamicin such as 200 µg/mL (Bryła and Trzcińska, 2015) and 250 µg/mL (Waberski et al., 2019). These considerations highlight the importance of establishing an appropriate antibiotic concentration in semen extender for different species. The significance of using an optimum dose was illustrated in a report on stallions, in which the use of 1 mg/mL gentamicin adversely affected sperm viability and motility during storage, and also impaired the VAP, VSL, and VCL (Aurich and Spergser, 2007). This shows that antibiotic concentration can really interfere with the sperm kinetic parameters as observed in the present study for peccary sperm VAP and subpopulations.

In parallel, we highlighted the effectiveness of streptomycin-penicillin combination at both the concentrations tested, which not only controlled bacterial growth, but also eliminated the microorganisms in some peccary semen samples stored under chilled conditions. This combination has been added to semen extenders since the 1950s in various species including bovine (Almquist, 1951), ovine (Moustacas et al., 2010), and equine (Dean et al., 2012), despite reports of resistance that date decades ago (Alford, 1953). Its use in refrigerated semen is reported at concentrations that vary between species and range from 38-105 µg/mL (in combination with 0.315 µg/ml amphotericin) in stallions (Dean et al., 2012) to 1 mg/mL - 1000 IU/mL in dogs (Lopes et al., 2009), which is the concentration used currently for the majority of domestic species. The effectiveness of the drug combination is related to their synergistic mechanism of action, which provides a broad spectrum of action and bactericidal potential. Streptomycin is an aminoglycoside antibiotic with gentamicin-like action (Luzzatto et al., 1968), and penicillin is a β-lactams antibiotic which interferes bacterial cell membrane synthesis, causing lysis, and cell death (Waxman and Strominger, 1983).

Streptomycin-penicillin, however, especially at highest concentrations (2 mg/mL – 2000 IU/mL), impaired some peccary sperm parameters as membrane integrity, and motility features as VAP and fast sperm subpopulation. One possible explanation for this is that bactericidal antibiotics (quinolones, aminoglycosides, and β-lactams) cause mitochondrial dysfunction and overproduction of reactive oxygen species (ROS) in mammalian cells, which leads to oxidative damage to DNA, proteins, and membrane lipids (Kalghatgi et al., 2013). Similar results were observed in bulls, where the use of 4000 IU/mL penicillin affected sperm motility, while streptomycin, at doses of up to 8 mg/mL had no effect on this parameter (Sykes and Mixner, 1951). In fact, the toxic effect of the drugs may be related to the variable sensitivity of sperm from different species, since a 10 × higher than standard concentration (10 mg/mL - 10,000 IU/mL) of streptomycin-penicillin was used in the extender for cryopreservation of semen from wild canids (*Canis lupus and C. lupus baileyi*), with no negative effects (Zindl et al., 2006).

Despite its reported antimicrobial potential (Kumar et al., 2019), no bacterial control was noted with the extender containing only *Aloe vera* gel. Moreover, no synergistic antibacterial effect among the gel and the antibiotics was evidenced. On the contrary, the bacterial load of the extenders containing only *Aloe vera* gel or egg yolk was similar. In fact, the *Aloe vera* metabolites associated with antibacterial activity as the anthraquinones, the glucomannan and the acemannan (Maan et al., 2018), can be influenced by several factors such as seasonality, rainfall, radiation, temperature, level of nutrients and water, age of the plant, among others (Gobbo-Neto and Lopes, 2007), which are inherent and vary according to the place of study. In this sense, variability in *Aloe vera* gel metabolites could also be a reason for the lower effectiveness of the extender containing the gel for preserving peccary sperm membrane integrity and mitochondrial activity when compared to the extenders containing egg yolk. These findings evidence the lack of standardization for the use of *Aloe vera* gel as a component of semen extenders, highlighting the needs for its chemical characterization (Farias et al., 2019).

At general, the bacterial load did not appear to affect the quality of the diluted peccary semen, which showed good results even in the control groups. However, in addition to the bacterial load, the deleterious effects on sperms also depend on the type of bacteria, incubation period, and temperature (Bonet et al., 2018). Furthermore, we emphasize that the bacterial load was not fully eliminated by the antibiotics used in this study. In this sense, the search for other concentrations of antibiotics or even for alternative drugs (Santos and Silva, 2020) should be directed towards providing effective protocols for bacterial control during semen storage.

Interestingly, the simple addition of the media to fresh semen from collared peccaries led to a reduction in sperm motility, regardless of the extender used. This has already been reported by our team in a previous work on the cooling of peccary semen, in which fresh samples that had 97% motility dropped to values between 60 and 70% after dilution in Tris containing yolk or *aloe vera* (Souza et al., 2016). Since temperature, pH and osmolarity of extenders were previously determined for the species (Souza et al., 2016), we speculate that further adjustments in extender composition or even in semen dilution and storage temperature are needed, especially as this effect is not commonly observed in other mammals. Peccary semen processing requires further investigations, especially to improve the sperm longevity for various days like for domestic swine (Menezes et al., 2020).

## Conclusion

In conclusion, we suggest the use of a Tris-egg yolk extender supplemented of 70 µg/mL gentamicin for the storage of collared peccary semen at 5ºC, to control bacterial load and maintain sperm longevity for 36 h. This study contributes an improved protocol for peccary semen storage by identifying antibiotics suitable for use in the short-time storage extenders, which may be safely used in assisted reproductive technologies (ART) such as in vitro fertilization (IVF) or artificial insemination (AI).
